# Incidence, severity and potential pathomechanism of immune‐related pruritus in melanoma patients undergoing immune checkpoint inhibitor therapy

**DOI:** 10.1111/ddg.70048

**Published:** 2026-03-25

**Authors:** Andrea Roggo, Nikolina Ivanovic, Patrick Turko, Lukas Kraehenbuehl, Mitchell Levesque, Reinhard Dummer, Barbara Meier‐Schiesser

**Affiliations:** ^1^ Department of Dermatology University Hospital Zurich University of Zurich, Switzerland; ^2^ Department of Dermatology Kantonsspital Aarau Aarau Switzerland

**Keywords:** Combination therapy, eosinophilic granulocytes, immune checkpoint inhibitors, immune‐related pruritus, melanoma, topical steroids

## Abstract

**Background and objectives:**

Pruritus is a frequent cutaneous immune‐related adverse event (irAE) associated with immune checkpoint inhibitors (ICI), impacting patients' quality of life. This study investigates the incidence, severity and management of pruritus in melanoma patients treated with ICI.

**Patients and methods:**

Clinical data from 461 melanoma patients at the University Hospital of Zurich were analyzed in a retrospective, monocentric study. Patients received ICI either as combination therapy (ipilimumab and nivolumab) or monotherapy (nivolumab or pembrolizumab).

**Results:**

Immune‐related pruritus (irPruritus) was associated with increased overall survival. It affected 23.0 % of patients (106/461), with a significantly higher frequency and earlier onset observed in combination therapy compared to monotherapy (*p* < 0.001). A significant, pronounced increase in eosinophil counts was noted from baseline to onset of irPruritus. Topical steroids proved effective in 90.9 % of cases. Additionally, irPruritus was significantly correlated with both irFatigue (*p* < 0.01) and irRash (*p* < 0.001).

**Conclusions:**

Pruritus is a common side effect of ICI therapy. Its correlation with irFatigue and irRash highlights the broader systemic impact impairing patients’ quality of life. Further prospective studies are needed to elucidate the underlying mechanisms such as involvement of eosinophils and identify optimal treatment strategies while maintaining ICI effectiveness.

## INTRODUCTION

Malignant melanoma, one of the most frequent cancers worldwide with a high mortality rate, represents a substantial health burden on a global scale.^1^ In the last two decades, immune checkpoint inhibition (ICI) using e.g. the anti‐PD‐1 antibodies pembrolizumab or nivolumab, or the anti‐CTLA‐4 antibody ipilimumab, either alone or in combination, have significantly improved overall survival (OS) in metastatic melanoma.^2^ Programmed cell death protein 1 (PD‐1) and cytotoxic T‐lymphocyte‐associated antigen 4 (CTLA‐4) are co‐inhibitory immune checkpoint receptors expressed on activated T cells that downregulate the immune response.^3^ They play a key role in maintaining the body's self‐tolerance and controlling inflammation in normal tissue.^3^

CTLA‐4 primarily functions in the early stages of T‐cell activation, where it reduces the activation of naive and memory T cells to prevent overactivation. Its inhibition leads to a broader immune response through a greater diversity of T‐cell receptors and increased tumor infiltration.^4^ In contrast, PD‐1 is essential in modulating immune responses in peripheral tissues, to avoid autoimmunity. PD‐1 blockade increases the number of T cells in the tumor without changing the diversity of T‐cell receptors in the rest of the body.^5^ By inhibiting T‐cell activity in inflamed or cancerous tissues, PD‐1 and CTLA‐4 can inadvertently help tumor cells evade immune detection.^6^ The combination of PD‐1 and CTLA‐4 inhibitors in metastatic melanoma has been shown to have a synergistic effect, with nivolumab and ipilimumab being used as a combination therapy.^7^

These therapies demonstrate high efficacy but are frequently associated with immune‐related adverse events (irAEs) in different organs such as skin, gastrointestinal tract, endocrine glands, lungs, and others.^8^, ^9^ The occurrence of irAEs has been shown to be associated with increased OS.^10^ Therapy with PD‐1 inhibitors most commonly is associated with mild to moderate irAE. Toxicity grade 3 or 4 as defined by the *Common Terminology Criteria for Adverse Events* (CTCAE) only occurs in 23 %.^9^ In contrast, grade 3–4 toxicity occurs significantly more frequently with combination therapy using ipilimumab and nivolumab (59 %).^9^ Early diagnosis and treatment are essential to prevent long‐term damage to quality of life for cancer patients. For the treatment of specific irAEs, guidelines recommend using corticosteroids and immunosuppressive agents.^11^, ^12^, ^13^ ICI therapy should be discontinued for toxicity grade 3 or higher.^14^, ^15^

Dermatological toxicities, affecting more than one‐third of patients, are the most prevalent irAEs associated with ICI.^16^ The most common dermatological toxicities are generally reported as rash (irRash) and pruritus (irPruritus).^16^ Also commonly reported are lichenoid irAEs, maculopapular rashes, eczematiform rashes, and vitiligo‐like depigmentation in metastatic melanoma, amongst others.^17^ Pruritus or itching is one of the most common cutaneous side effects related to ICI therapy, occurring in 11 % to 47 % of cases. It can result in increased morbidity in patients, and the effects on their quality of life can thereby have a negative impact on the course of treatment.^16^, ^18^, ^19^ Despite the clinical relevance of pruritus as a potential irAE^19^, its clinical characterization remains limited.^18^ Treatment recommendations include basic measures such as using fragrance‐free skin moisturizers.^20^ Additionally, mild‐ to medium potency topical corticosteroids (TCS) can be applied along with systemic administration of non‐sedating antihistamines. In severe or refractory cases, GABA analogs replace anti‐histamines.^20^ Limited evidence is available for other systemic treatments such as dupilumab,^21^ omalizumab,^22^ or NK1‐antagonists.^23^ Furthermore, phototherapy with narrowband ultraviolet B (UVB) can be attempted if feasible.^24^ However, as the detailed pathomechanism leading to irPruritus is not yet fully understood, no targeted intervention has been validated.

This retrospective study aims to systematically analyze the association between ICI and the occurrence of irPruritus after therapy initiation in metastatic melanoma patients. To better understand this challenging adverse event, we analyze the clinical course, potential predisposing factors, and the correlation with treatment response. The insights gained should provide a base for optimized management of irPruritus whilst maintaining efficacy of life‐saving ICI.^25^

## PATIENTS AND METHODS

This retrospective cohort study examined patients with metastatic melanoma treated with pembrolizumab, nivolumab, or a combination of nivolumab and ipilimumab at the Department of Dermatology, University Hospital Zurich (USZ), a tertiary academic cancer center. Data collection involved analyzing records of metastatic melanoma patients treated between 2014 and 2022. Patients were identified from the EUMELAREG database, filtering for the prespecified therapy regimens. Clinical data was extracted from medical records in the KISIM patient data system.

Inclusion criteria were a histologically confirmed melanoma diagnosis, documented treatment with pembrolizumab, nivolumab or ipilimumab and nivolumab, age over 18 years and availability of clinical data. Patients who declined USZ general research consent were excluded from the study. Extracted information included patient characteristics (age, sex, date of death as of 10^th^ of May 2025, medical history including pruritic diseases), tumor characteristics (TNM stages, localization, mutation status), metastatic melanoma therapy details (type of therapy, duration of therapy, number of infusions) and immune therapy related adverse events, and pruritus characteristics (onset, localization, severity, therapy). Eosinophil counts were measured at baseline prior to treatment initiation and at the onset of irPruritus. For patients without immune‐related pruritus (irP), eosinophil counts were extracted at infusion time points corresponding to the median onset of irP in each treatment group (ipilimumab/nivolumab: infusion 2; nivolumab: infusion 7; pembrolizumab: infusion 10). The severity of irPruritus and irRash was assessed using CTCAE staging. TCS was classified as mild, moderately potent, potent and very potent. In patients who received multiple lines of therapy, second‐line treatment was included only if more than 6 months had elapsed between treatment courses. Data was collected in the program Microsoft Excel (Microsoft Corporation, Seattle, WA, USA).

Statistical testing was performed with R (version 4.3.1) using Welch's t‐test for numeric variables and Chi‐squared test of independence for categorical variables. Time‐to‐event data was investigated with a multivariate Cox proportional hazard analysis. Profiling of relative eosinophilic counts in patients with and without irPruritus was performed with a generalized linear mixed model using the glmmTMB package. The model included timepoint (baseline vs follow‐up), irPruritus status (yes/no) and their interaction as fixed effects, with random intercepts for each patient. p‐values ≤ 0.05 were considered statistically significant.

### Ethical approval

This project was reviewed and approved by the Ethics Committee of the Canton of Zurich. All patients provided written informed consent for the publication of their case details.

## RESULTS

### Combination therapy more frequently causes irPruritus compared to monotherapy

A total of 461 patients were included in this study (Table 1). The mean age was 62.6 years (standard deviation [SD] 15.0), with 284 (61.6 %) male and 177 (38.4 %) female patients. Among those who developed pruritus as an irAE (irPruritus), 41 (38.7 %) were female, and 65 (61.6 %) were male, with a mean age of 63 years (SD 15.2). In the overall cohort, most melanomas were stage IV (58.3 %). A considerable proportion of cases had mutations in the *NRAS* (21.7 %) and *BRAF* (34.3 %) genes. No correlation was found between incidence of irPruritus and clinical parameters such as age, gender, staging, tumor mutations and medical history (*p* > 0.05). Primary melanomas were most frequently located on the extremities (37.8 %), followed by the trunk (27.3 %), head/neck (21.5 %), and mucous membranes (2.2 %). The differences between pruritus localizations were not statistically significant (*p* = 0.49). There was also no significant correlation with preceding pruritic diseases, non‐melanoma malignant tumors, renal failure, diabetes or neuronal diseases during ICI therapy. 199 patients (43.3 %) received pembrolizumab, 148 (32.1 %) nivolumab, and 114 (24.7 %) the combination of ipilimumab and nivolumab. The average duration of therapy was 35.2 weeks (SD 36.3) with an average of 12.0 infusions (SD 11.0).

**TABLE 1:** Patient characteristics. Overview of clinical parameters in the investigational cohort and stratified by immune‐related pruritus (irP). Statistical analyses included Welch's t‐test (continuous variables) and the chi‐square test of independence (categorical variables).

	All patients (n = 461)	patients without pruritus (n = 355)	patients with pruritus (n = 106)	*p*‐value
Sex, n (%)				0.94
male	284 (61.6)	219 (61.3)	65 (61.6)	
female	177 (38.4)	136 (38.3)	41 (38.7)	
Age, mean years (SD)	63 (15.0)	62 (15.6)	64 (15.2)	0.42
Medical history, n (%)				
malignancy	147 (31.9)	116 (32.7)	31 (29.2)	0.51
itchy disease	129 (28.0)	97 (27.3)	32 (30.2)	0.56
neuronal disorder	76 (16.5)	56 (15.8)	20 (18.9)	0.45
kidney failure	54 (11.7)	38 (10.7)	16 (15.1)	0.22
diabetes	53 (11.5)	37 (10.4)	16 (15.1)	0.18
Staging, n (%)				0.68
II	16 (3.4)	11 (3.2)	5 (4.6)	
III	176 (38.3)	136 (38.3)	40 (37.6)	
IV	269 (58.3)	208 (58.6)	61 (57.5)	
Mutation, n (%)				
NRAS	100 (21.7)	78 (22.0)	22 (20.8)	0.79
BRAF	158 (34.3)	130 (36.6)	28 (26.4)	0.05
Localization, n (%)				0.49
extremity	174 (37.7)	139 (39.2)	35 (33.0)	
trunk	126 (27.3)	91 (25.6)	35 (33.0)	
head/neck	99 (21.5)	75 (21.1)	24 (22.6)	
mucosa	9 (2.0)	8 (2.3)	2 (1.9)	
unknown primarius	53 (11.5)	42 (11.8)	11 (10.4)	
Therapy, n (%)				0.03
pembrolizumab	199 (43.3)	155 (43.7)	44 (41.5)	
nivolumab	148 (32.1)	122 (34.4)	26 (24.5)	
ipilimumab/nivolumab	114 (24.7)	78 (22.0)	36 (34.0)	
Treatment weeks, mean (SD)	35 (36.3)	34 (36.5)	40 (35.9)	0.19
Infusions, n (SD)	12 (11.0)	12 (11.2)	13 (10.6)	0.28

Abbr.: SD, standard deviation

To better understand the therapeutic relevance of irAEs, we investigated their relation to the OS. irPruritus was non‐significantly associated with improved OS (HR: 0.82, *p* = 0.35, multivariable Cox regression) (Figure 1a). Among the reported irAEs, irEdema, irHepatitis, irMyasthenia, irMyositis, irPneumonia, and irColitis/Gastritis showed a tendency toward reduced OS (online supplementary Figure S1a–e). In contrast, irRash, irPancreatitis, irArthritis, irFatigue, irHypothyreosis, irVitiligo, irNephritis, irXerostomia, irKeratitis sicca, and irKeratosis were associated with increased OS (online supplementary Figure S1f–o). Among patients with irPruritus (n = 106), prevalence was significantly higher with combination therapy (31.6 %) compared to monotherapy with nivolumab (17.6 %) or pembrolizumab (22.1 %, *p* = 0.03) (Table 2). Notably, irPruritus occurred significantly earlier under combination therapy, on average after 1.6 infusions (SD 0.8) compared to pembrolizumab after 7.4 infusions (SD 7.1) and nivolumab after 8.8 infusions (SD 5.2) (*p* < 0.001). There was no significant difference in the characteristics of irPruritus between the three treatment regimens.

**FIGURE 1:**
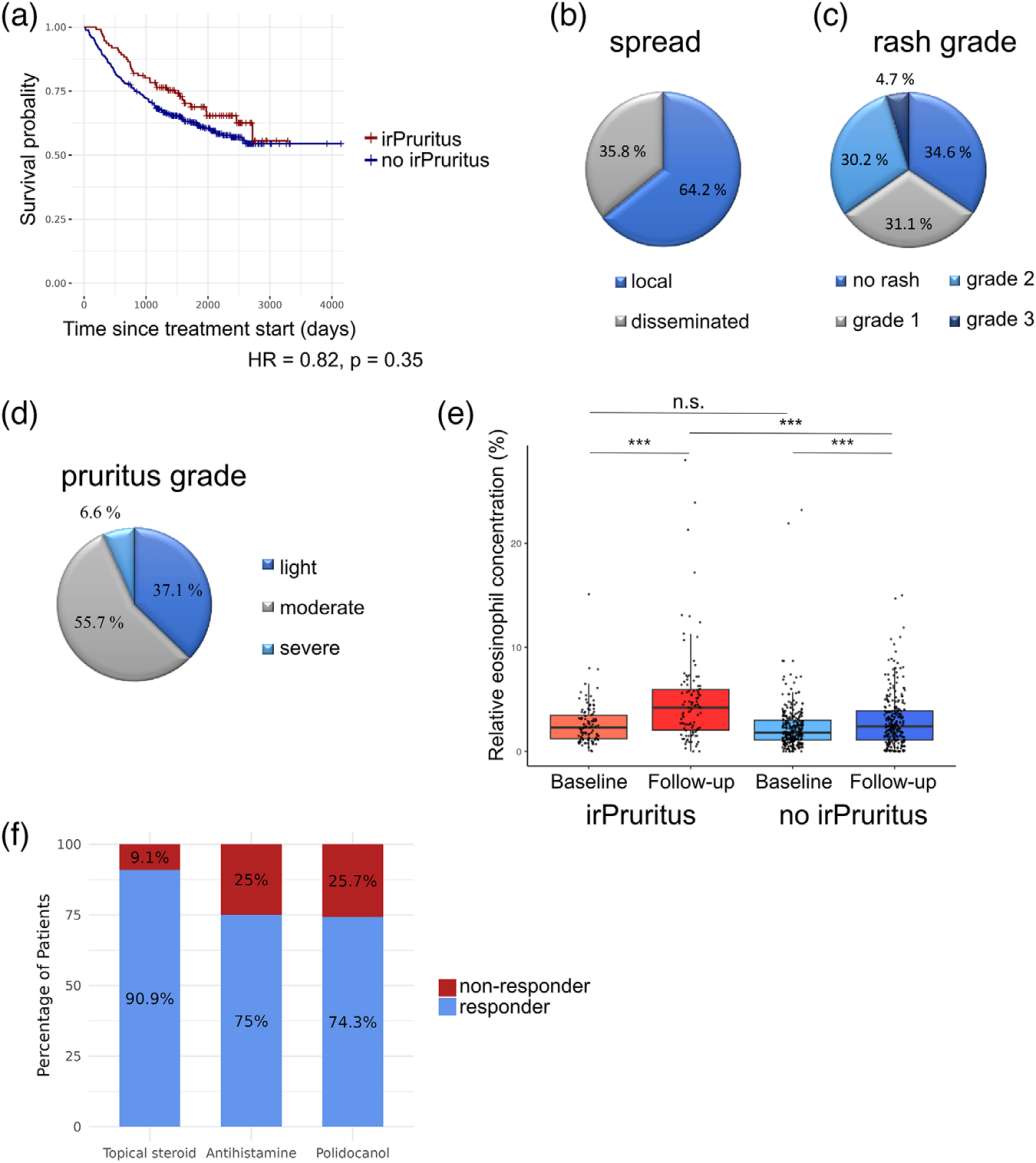
Clinical characteristics of immune‐related pruritus (irPruritus) and its association with eosinophil counts. (a) Kaplan–Meier curve showing overall survival probability in patients with (n = 106) and without irPruritus (n = 355). Statistical analysis was performed using a multivariable Cox proportional hazards model including all evaluated immune‐related adverse events. Pie charts display descriptive statistics among patients with reported irP (n = 106), including (b) distribution, (c) rash grade associated with pruritus, and (d) pruritus severity. (e) Boxplots showing relative eosinophil counts. Values were extracted prior to treatment initiation (baseline), at the onset of irPruritus (follow‐up irPruritus group), or at infusion time points corresponding to the median onset of irPruritus per therapy (follow‐up non‐irP group; Table 2). Statistical analysis was performed using a generalized linear mixed model. (f) Bar graph illustrating the number of successful outcomes per therapy relative to the number of treated cases. Absolute numbers and relative percentages are indicated. *p < 0.05; **p < 0.01; ****p < 0.001

**TABLE 2:** Pruritus characteristics stratified by received therapy. Statistical analyses included Welch's t‐test (continuous variables), the chi‐square test of independence (categorical variables), and Cox regression analysis (time‐dependent variables).

	Ipilimumab/nivolumab (n = 36)	Pembrolizumab (n = 44)	Nivolumab (n = 26)	p‐value
Pruritus prevalence, n (%)	36 (31.6)	44 (22.1)	26 (17.6)	0.03
Onset of pruritus, median infusion (SD)	1.0 (0.8)	9 (7.1)	6 (5.2)	< 0.001
Pruritus characteristics, mean (SD)				
rash	0.7 (0.5)	0.6 (0.5)	0.7 (0.5)	0.4
grade	1.1 (0.9)	0.8 (0.8)	1.0 (0.9)	0.3
severity	1.9 (0.5)	1.6 (0.6)	1.6 (0.6)	0.1

*Abbr*.: SD, standard deviation

Detailed characterization of irPruritus (n = 106) revealed local manifestation of pruritus in 64.2 % (n = 68) and a disseminated pattern in 35.8 % (n = 38) (Figure 1b). IrRash occurred simultaneously in 70 patients (66.0 %). Within this cohort, all severity grades were reported with most patients reporting rash grade 1 (n = 33, 31.1 %), followed by grade 2 (n = 32, 30.2 %) and grade 3 (n = 5, 4.7 %) (Figure 1c). No classification of irRash was documented in two cases. To better understand the severity of the symptom, irPruritus was categorized as mild, moderate or severe according to CTCAE grades.^9^ Among all patients reporting irPruritus, 59 (55.7 %) developed moderate itching, 40 (37.1 %) developed mild itching and seven (6.6 %) developed severe itching (Figure 1d). One patient had to discontinue therapy due to severe irPruritus.

### Eosinophils are elevated during irPruritus

To investigate differences in laboratory parameters between patients with and without irPruritus, blood counts were analyzed. For patients without irPruritus, the timepoint was matched to the median onset of irPruritus per therapy regimen (Table 2). At baseline (prior to the first infusion), eosinophil levels did not differ significantly between patients who later developed irPruritus and those who did not (*p* = 0.4, generalized linear mixed model) (Figure 1e). During therapy, both groups showed a significant increase in eosinophil counts (*p* < 0.001 for each, generalized linear mixed model) (Figure 1e). Notably, this increase was more pronounced in patients with irPruritus, whose eosinophil levels rose from 2.3 % at baseline to 4.2 % at the time of symptom onset.

### irPruritus responds to topical steroids

In our cohort, irPruritus‐specific therapy included TCS (n = 55), antihistamines (n = 20) and topical application of polidocanol (n = 70) (Figure 1f). TCS were effective in 90.9 % of patients, followed by antihistamines (75.0 %), and polidocanol (74.3 %). The TCS used were classified as moderately potent (class II: clobetasone butyrate 0.05 %, triamcinolone acetonide 0.1 %), potent (class III: mometasone furoate 0.1 %, betamethasone valerate 0.1 %, betamethasone dipropionate 0.05 %), and very potent (class IV: clobetasol propionate 0.05 %). Class III was effective in 38/41 (93.2 %) of patients, Class IV in 9/10 (90.0 %), and Class II in 3/4 (75.0 %) (online supplementary Table S1).

### irPruritus is associated with chronic irFatigue and frequently occurs with an irRash

ICI can cause a range of irAEs, including irPruritus (23.0 %), irRash (25.2 %), irFatigue (21.9 %), irHepatitis (15.8 %), and irColitis/Gastritis (16.7 %) (Table 3). To examine potential correlations, we explored the relationship between irPruritus and other irAEs. Among patients with irPruritus, 74 (69.2 %) developed irRash and 34 (32.1 %) experienced irFatigue. Both irFatigue and irRash showed a significant correlation with irPruritus (*p* < 0.001 and *p* < 0.01 respectively). No other irAEs showed an association with irPruritus.

**TABLE 3:** Prevalence of immune‐related adverse events and their association with immune‐related pruritus (irP). Statistical analysis was performed using logistic regression.

	All patients (n = 461)	Patients without pruritus (n = 355)	Patients with pruritus (n = 106)	p‐value
***Adverse event, n (%)***				
irRash	116 (25.2)	42 (11.8)	74 (69.2)	< 0.001
irFatigue	101 (21.9)	67 (18.9)	34 (32.1)	< 0.01
irHepatitis	73 (15.8)	53 (14.9)	20 (18.9)	0.6
irColitis/Gastritis	77 (16.7)	58 (16.3)	19 (17.9)	0.7
irHypothyreosis	71 (15.6)	52 (14.6)	20 (18.9)	0.4
irXerostomia	28 (6.1)	23 (6.5)	5 (4.7)	0.5
irPneumonia	27 (5.9)	22 (6.2)	5 (4.7)	0.3
irPancreatitis	24 (5.2)	21 (5.9)	3 (2.8)	0.1
irEdema	12 (2.6)	10 (2.8)	2 (1.9)	0.2
irVitiligo	11 (2.4)	9 (2.5)	2 (1.9)	0.8
irMyasthenia/Myositis	9 (2.0)	7 (2.0)	2 (1.9)	0.7
irNephritis	6 (1.3)	4 (1.1)	2 (1.9)	0.2
irKeratitis sicca	8 (1.7)	8 (2.3)	0.0	0.1
irKeratosis	3 (0.7)	3 (0.8)	0.0	0.1

## DISCUSSION

Our study highlights the relevance of irPruritus during treatment with ICI, and in particular with combination therapy. The incidence of irPruritus during monotherapy (nivolumab 17.6 %, pembrolizumab 22.1 %) was significantly lower compared to combination therapy with ipilimumab and nivolumab (31.6 %). This is in line with previous investigations in smaller cohorts.^16^, ^18^, ^26^ An even higher rate of 33.2 % was observed under combination therapy in 104 of 313 patients.^27^, ^28^

IrAEs resulting from immune system overactivation may reflect effective immune stimulation under ICI.^29^ Benign, low grade irAE that can be managed without discontinuing ICI treatment are therefore be considered potential indicators of a favorable therapeutic response.^29^, ^30^ In our cohort, irPruritus, irXerostomia or irKeratitis sicca were associated with prolonged OS. Supporting this observation, irPruritus was mostly low‐grade and only led to therapy discontinuation in one out of 461 patients in our cohort. Other studies have shown that grade 3/4 irPruritus occurred in less than 1 % with nivolumab monotherapy.^31^ However, given the high prevalence of grade 1/2 irPruritus and its impact on patient's quality of life, a better understanding of appropriate treatment is needed. In our cohort, TCS as irPruritus therapy were effective in 90.9 % of patients, while antihistamines and topical polidocanol were less effective. Class III TCS were used most frequently, with an efficacy of 93.2 %. The choice of TCS potency should take into account the body site, as efficacy depends on factors such as epidermal thickness, barrier function, affected skin area, and formulation base.^32^ Given the low absorption of short‐term treatment with locally applied TCS, in particular patients with localized, low‐grade irPruritus can profit and the risk of impairing ICI efficacy remains relatively low. However, with wide‐spread application of TCS, systemic absorption exceeding 20 mg prednisone equivalent is possible.^32^ In summary, it also confirms that there is an unmet clinical need for better management of pruritus in those patients not benefitting from TCS, oral antihistamine, and topical polidocanol. In addition to the pruritus‐specific therapy applied in our cohort, GABA‐analogues may be considered for refractory cases. Furthermore, growing evidence supports the use of IL4R blockade with dupilumab.^21^

The exact cause of pruritus occurring under ICI has not been elucidated to date. In our study, age, sex, medical history, melanoma‐staging, and location were not related to the prevalence of irPruritus. Similar results have been published in a smaller cohort of patients with melanoma.^18^ ICI therapy for other types of cancers have a similar side‐effect profile with a pruritus‐prevalence ranging from 10 % to 14 %–47 %.^18^, ^33^ These results suggest that irPruritus is a direct consequence of ICI therapy and not attributable to melanoma.

In this study, an increased relative count of eosinophils in the blood of pruritic patients was observed compared to their counts before starting treatment. The role of eosinophils in ICI therapy is poorly understood. They have been proposed as a predictive biomarker, associated with improved outcomes and irAEs.^34^ A hypothesis has been put forward regarding a direct association between CTLA4 inhibitors and irPruritus, attributed to the resulting increased immune activity in the skin.^35^, ^36^ Indeed, histological examination of skin biopsies from eight out of nine patients who develop irPruritus and irRash during ipilimumab treatment shows the presence of superficial, perivascular, CD4^+^‐dominant T‐cell infiltrates, accompanied by eosinophils in the dermis.^35^ Consistent with our findings, an increase in eosinophil levels was noted in patients' blood at the onset of irPruritus.^6^, ^18^, ^35^ Future studies should consider performing histological examinations of the skin in patients with irPruritus and an assessment of eosinophil levels before and during the onset of irPruritus, and comparing findings between the skin and blood. Another study revealed that irPruritus without rash was the most common grade 2 or higher cutaneous adverse event in a prospectively recruited cohort. Increased levels of IL‐4 were observed in both the blood and skin of patients affected by irPruritus. Furthermore, the number of eosinophils in pruritic skin without rash was significantly higher than in control subjects.^37^ These findings provide strong support for targeted interventions, such as inhibiting IL4R with dupilumab.^21^ Notably, in our cohort, patients with and without irPruritus had equal eosinophilic counts before starting the treatment, suggesting that eosinophil counts cannot be used as predictive biomarker for irPruritus.

Analysis of concomitant irAEs revealed that irPruritus was significantly associated with irFatigue and irRash (*p* < 0.01 and *p* < 0.001, respectively) in our patient cohort. Previous studies report that irFatigue occurs in 15 %–35 % and irRash in 15 %–40 % of metastatic melanoma patients treated with ICIs.^5^, ^26^, ^28^ The correlation between irPruritus and irFatigue may stem from the impact of pruritus on patients’ sleep, however systemic inflammation caused by ICI may also contribute to pruritus. As a symptom of irRash, irPruritus is mediated by the stimulation of polymodal C nerve fibers in the skin. It can be triggered directly by chemical and mechanical stimuli or indirectly through interactions with mast cells, T lymphocytes, or eosinophils that release pruritogenic mediators. Consequently, irPruritus may occur with or without the presence of irRash.^38^ This study adds to the existing body of evidence by including a large patient cohort; however, its monocentric and retrospective design represents a limitation, underscoring the need for complementary prospective and mechanistic studies. These findings highlight the importance of systematic clinical follow‐up in patients receiving immune checkpoint inhibitor (ICI)‐based cancer therapy, enabling earlier and more individualized interventions to maintain both ICI treatment and patients’ quality of life.

## CONFLICT OF INTEREST STATEMENT

M.L. has received unrelated research support from Roche, Novartis, Bacoba, Scailyte, and Oncobit and serves as an occasional scientific advisor on unrelated projects for Roche, Leica, Bacoba, Oncobit, and Scailyte. R.D. reports intermittent, project‐focused consulting and/or advisory relationships with Novartis, Merck Sharp & Dohme (MSD), Bristol‐Myers Squibb (BMS), Roche, Amgen, Takeda, Kyowa Kirin, Immunocore, Pierre Fabre, Sun Pharma, Sanofi, Catalym, Second Genome, Regeneron, Alligator, T3 Pharma, MaxiVAX SA, Pfizer, Oncobit, and touchIME outside the submitted work. A.R., N.I., P.T., L.K., and B.M.S. declare no conflict of interest.

## Supporting information



Supplementary information
