# Supplementary material for: Incidence, severity and potential pathomechanism of immune‐related pruritus in melanoma patients undergoing immune checkpoint inhibitor therapy
Source: J Dtsch Dermatol Ges. 2026 Mar 25;24(11):1566–73. doi: 10.1111/ddg.70048 (PMC13749609; doi:10.1111/ddg.70048)
Supplement: Supplementary file 1 [file DDG-24-1566-s001.pdf]

[[List of supplementary Figures and Tables]]

Supplementary FIGURE S1 Overall survival across all reported immune-related adverse events. Statistical analysis was performed using a multivariable Cox proportional hazards model including all evaluated immune-related adverse events. Abbr.: HR, hazard ratio

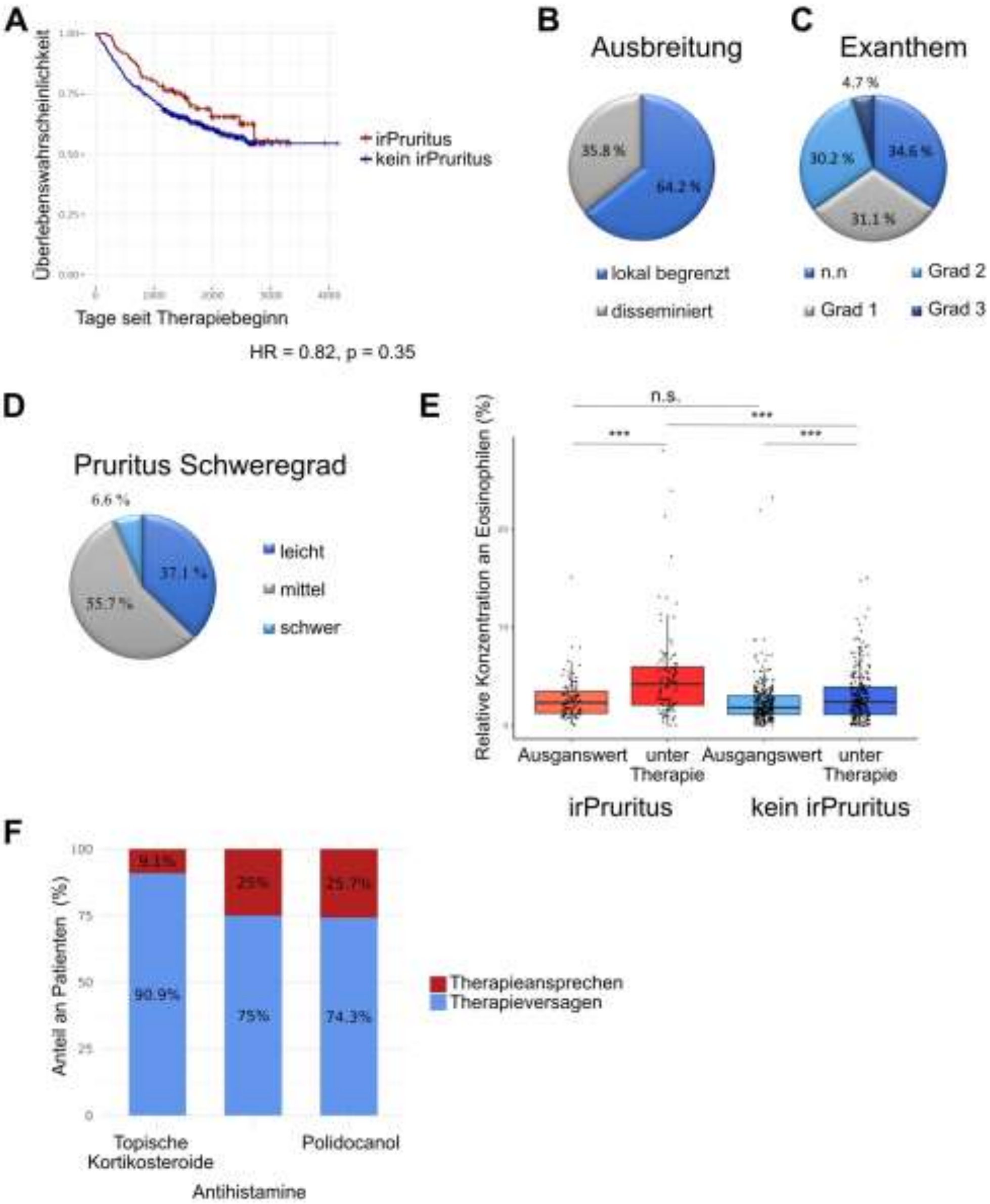
A
Überlebenswahrscheinlichkeit
irPruritus
kein irPruritus
Tage seit Therapiebeginn
HR = 0.82, p = 0.35
B
Ausbreitung
35.8 %
64.2 %
lokal begrenzt
disseminiert
C
Exanthem
4.7 %
30.2 %
34.6 %
31.1 %
n.n
Grad 2
Grad 1
Grad 3
D
Pruritus Schweregrad
6.6 %
37.1 %
55.7 %
leicht
mittel
schwer
E
Relative Konzentration an Eosinophilen (%)
n.s.
***
***
***
Ausganswert
unter Therapie
Ausgangswert
unter Therapie
irPruritus
kein irPruritus
F
Anteil an Patienten (%)
100
75
50
25
0
9.1%
25%
25.7%
90.9%
75%
74.3%
Therapieansprechen
Therapieversagen
Topische Kortikosteroide
Antihistamine
Polidocanol

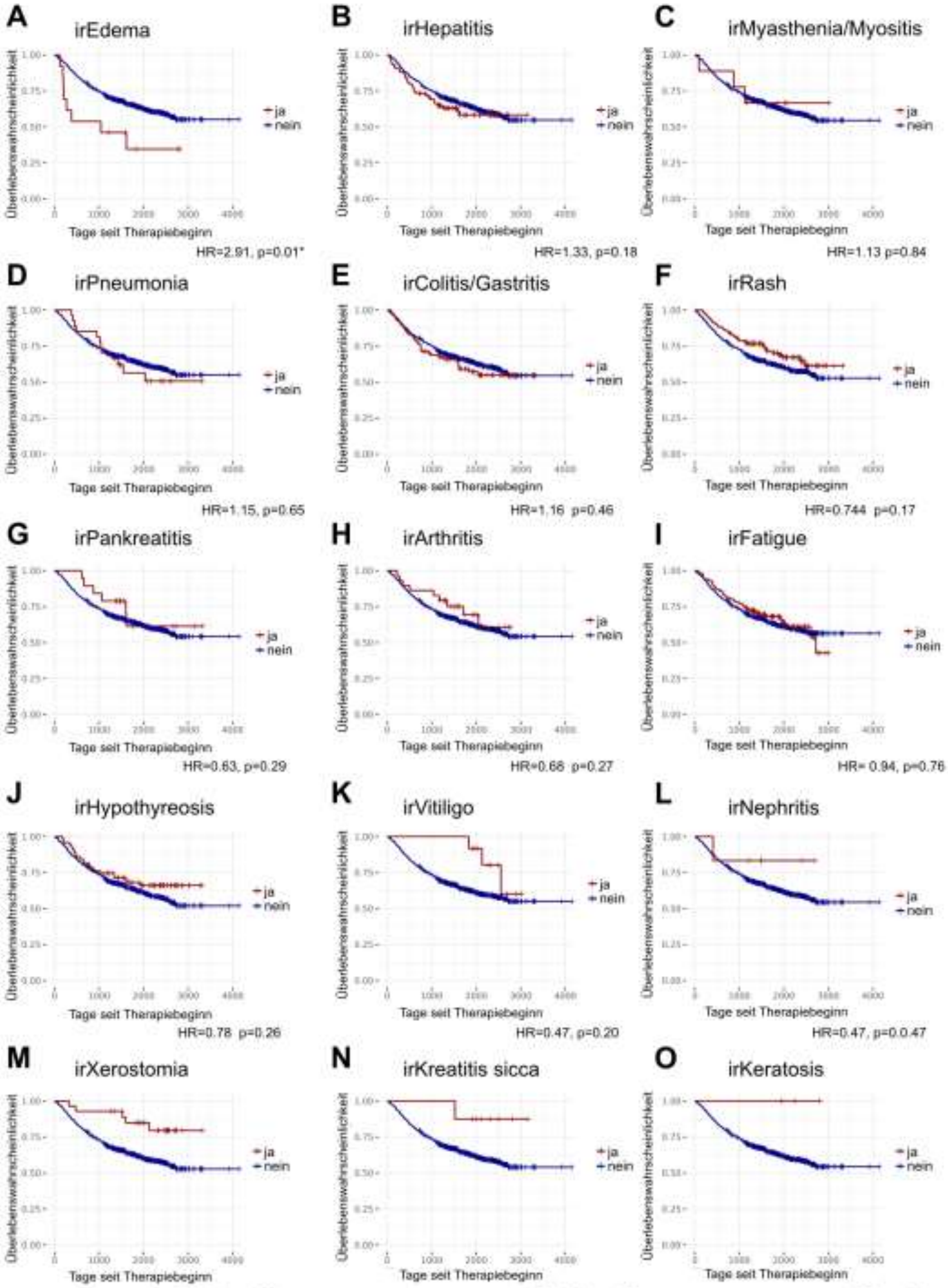
A
irEdema
HR=2.91, p=0.01*
B
irHepatitis
HR=1.33, p=0.18
C
irMyasthenia/Myositis
HR=1.13 p=0.84
D
irPneumonia
HR=1.15, p=0.65
E
irColitis/Gastritis
HR=1.16 p=0.46
F
irRash
HR=0.744 p=0.17
G
irPankreatitis
HR=0.63, p=0.29
H
irArthritis
HR=0.68 p=0.27
I
irFatigue
HR= 0.94, p=0.76
J
irHypothyreosis
HR=0.78 p=0.26
K
irVitiligo
HR=0.47, p=0.20
L
irNephritis
HR=0.47, p=0.0.47
M
irXerostomia
HR=0.34 p=0.03*
N
irKreatitis sicca
HR= 0.30, p=0.23
O
irKeratosis
HR=0, p=1.0
Überlebenswahrscheinlichkeit
Tage seit Therapiebeginn
ja
nein

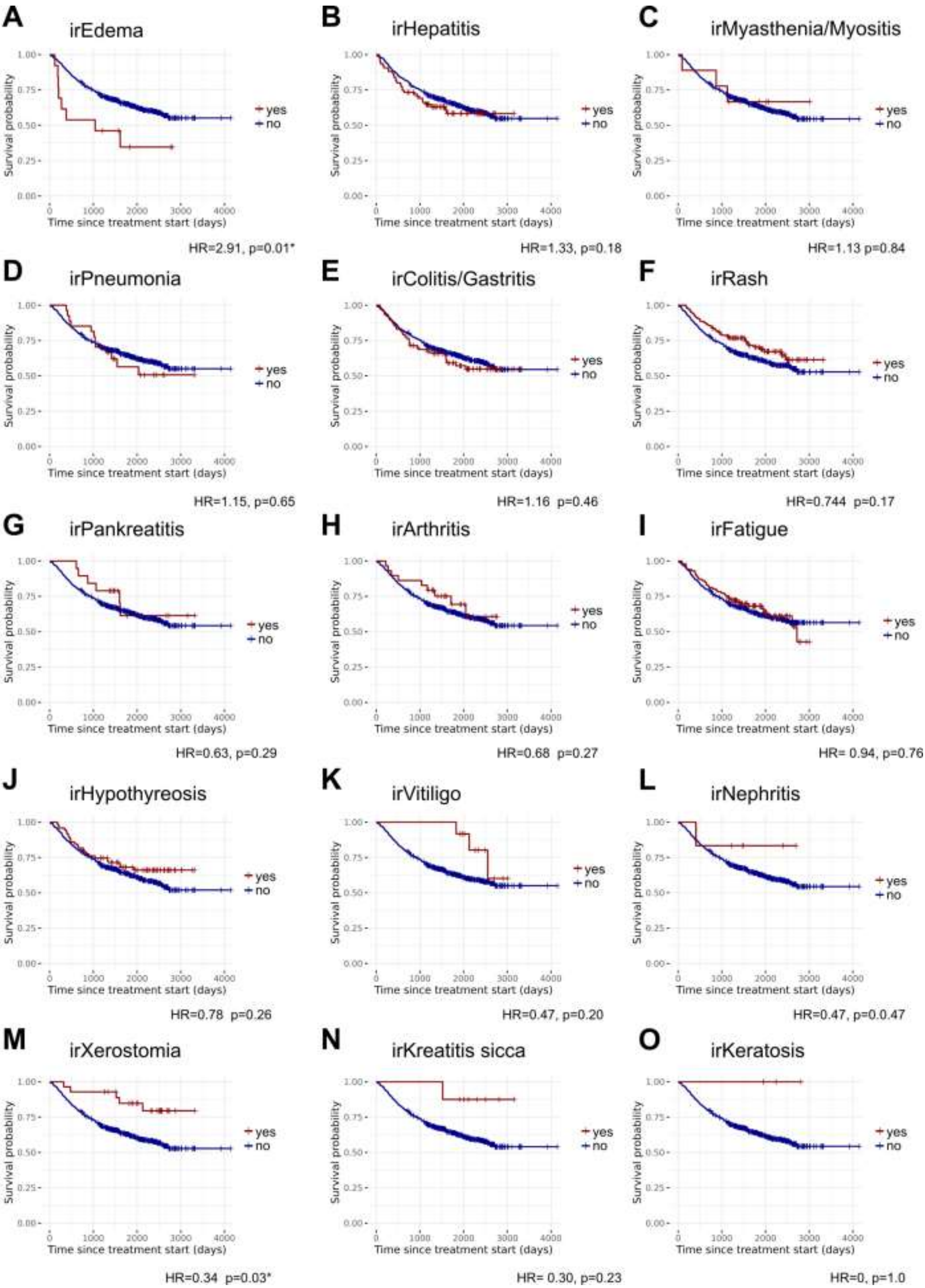


Supplementary TABLE S1 Frequency and success rate of topical corticosteroids (TCS) for the treatment of immune-related pruritus (irP) according to steroid potency.

Supplementary Table S1:

| | Responder | Non-responder |
|---|---|---|
| ***TCS-classification n (%)*** | | |
| I | 0 | 0 |
| II | 3 (75.0) | 1 (25.0) |
| III | 38 (92.7) | 3 (7.3) |
| IV | 9 (90.0) | 1 (10.0) |
